# One‒pot green synthesized protein‒based silver nanocluster as prooxidant biosensor

**DOI:** 10.3906/kim-2104-27

**Published:** 2021-10-19

**Authors:** Esin AKYÜZ

**Affiliations:** 1 Department of Chemistry, Faculty of Engineering, İstanbul University-Cerrahpaşa, İstanbul Turkey

**Keywords:** Prooxidant biosensor, silver nanocluster, chicken egg white, natural antioxidants, protein oxidation

## Abstract

In this study, silver nanoclusters as prooxidant biosensor were eco‒friendly synthesized using chicken egg white protein without any chemical reducing agents for measuring copper(II)-induced prooxidant activities of catechin, epicatechin, epigallocatechin gallate, resveratrol, gallic acid, chlorogenic acid, and rutin. The prooxidant activities were evaluated via measuring the absorption at 450 nm wavelength of the Cu(I)‒neocuproine chelate formed by extraction of protein-bound Cu(I) with neocuproine reagent. Accuracy was determined by evaluating recovery values of wine, grape and apple samples and the obtained values were between 97.2%‒98.9%. Intra-day precision and inter-day reproducibility experiments were studied with three different experiments in a day and three different days respectively. The obtained relative standard deviation values were 0.96% and 1.91%. The detection limit of the biosensor was found as 0.2 µM. The total prooxidant activities of fresh apple and grape fruits, apple and grape juices, and red wine were determined and the results obtained were compared with the findings of the carbonyl assay. In this study, a cheap, easily applicable, sensitive, and reproducible biosensor was developed. It was seen that it could be used in the measurement of the prooxidant activity of different food samples and give an idea about diet, healthy life, and nutrition.

## 1. Introduction

Copper is a vital and essential micro-bioelement for organism which required for the function of over 30 proteins, including ceruloplasmin, cytochrome c oxidase, tyrosinase, and superoxide dismutase. Also it was found mainly in the Cu(II) form in the biological systems, since in the presence of oxygen via Fenton‒type reaction Cu(I) is oxidized to Cu(II). The most reactive species, hydroxyl radicals release as a result of this reaction and induce “site-specific” oxidative damage to biological macromolecules [1,2]. The prooxidant activity is the capacity of reducing transition metal ions to their lower oxidation states by antioxidants, exciting the production of reactive species. Therefore, it is important to understand whether it is beneficial or harmful to health, depending on the amount of these compounds. 

As secondary metabolites derived in plants, phenolic exhibit various physiological properties such as antimicrobial, antiinflammatory, antioxidant, and antiallergenic [3]. Despite their antioxidative properties are well known, they may act as a prooxidant depending on some conditions such as high concentration, high pH, in the presence of redox‒active metal ion and biological material in the medium [4]. Furthermore, it has been noticed that prooxidant activity increases with increasing the number of free‒OH substitutions on the molecule structure [5]. In addition, since these compounds are used as food additives, it is very important to examine their antioxidant or prooxidant behavior depending on the conditions in which they are found.

In recent years, noble metal nanoclusters (NCs) have received extraordinary attention due to their many properties such as easy synthesis, subnanometer size, photostability, and biocompatibility [6]. Unfortunately, the most of silver nanocluster (AgNC) synthesises found in the literature contain NaBH_4_ as chemical reducing agent [7‒10] and there are rare biological reduction synthesis methods for AgNC [11,12]. Metal NCs were able to interact with each other and aggregate irreversibly to reduce their surface energy without stabilization [13]. In this context, metal NCs are biologically synthesized via the reduction of metal ions by the suitable reducing and stabilizing agents such as protein, peptide or nucleic acid where the thiol, carboxyl, and amine groups of biological molecules may be effective to stabilize NCs [7,10,14]. The NCs are able to act as sensitive probes with their optical response is highly dependent on the interaction with the organic scaffold. The application fields of different types of silver nanoclusters (AgNCs) have included iron [10], mercury [14,15] or biothiols [16] detection, and biological imaging [17].

Measuring prooxidant activity is a developing research area, required to be interested in with more work. Also it is needed to develop cheap, easily applicable, sensitive, and reproducible sensors. Existing assays in the literature are expensive especially in detecting radicals by electron spin resonance (ESR) spectroscopy, laborious, limited with respect to application area, and cause positive errors in results because of interferences, likewise in protein carbonyl assay. In our previous studies, we have developed new methods to overcome these disadvantages in the literature with the needs of this research area [18‒22].

In this study, chicken egg white protein directed silver nanocluster (CEW‒AgNC) biosensor, where the protein was used as both reducing and protecting agent, was prepared to detect prooxidant activity of phenolic compounds including in fruits, fruit juices, and red wine. In the method, it is thought that cuprous ions formed as a result of reduction of cupric ions by phenolic compounds are bound to thiol groups of protein in CEW‒AgNC surface. The protein bound cuprous ion which is the indicator of prooxidant activity, was determined colorimetrically by forming a Cu(I)‒neocuproine (Nc) complex with Nc reagent. Total prooxidant activities (TPAs) of fresh fruits, commercial fruit juices, and red wine were examined and statistically compared with the carbonyl assay. 

## 2. Materials and methods

### 2.1. Reagents and instrumentation

The following chemicals/reagents were supplied from the indicated sources: catechin (CAT), resveratrol (RES), epigallocatechin gallate (EGCG), epicatechin (ECAT), chlorogenic acid (CLA), and rutin (RT) from Sigma (Taufkirchen, Germany); tetrachloroauric acid (HAuCl_4_) and neocuproine (Nc) from Aldrich (Taufkirchen, Germany); gallic acid (GA), silver nitrate (AgNO_3_), sodium dihydrogen phosphate dihydrate (NaH_2_PO_4_.2H_2_O), ethanol (EtOH), and 2,4‒dinitrophenylhydrazine (DNPH) from Sigma‒Aldrich (Taufkirchen, Germany); ethylenediaminetetraacetic acid (EDTA) disodium salt and copper(II) sulfate from Fluka (Buchs, Switzerland); disodium hydrogen phosphate (Na_2_HPO_4_), hydrochloric acid (HCl) and sodium hydroxide (NaOH) from Riedel‒de Haën (Seelze, Germany). Fresh apple, apple juice, fresh grape, grape juice, and red wine were purchased from a local market. 

### 2.2. Preparation of solutions

Neocuproine (7.5 mM) and the stock standard antioxidant (10.0 mM) solutions were prepared in EtOH and diluted daily for experiments. Copper (2.0 mM), pH 7.4 phosphate buffer (0.5 M), and EDTA (0.1 M) solutions were prepared in distilled water. DNPH reagent was prepared in 0.2 N HCl solution. Two grams of fresh apple and grape were extracted using the microwave-assisted extraction technique as described in our previous study after all samples were washed with water and cut into small pieces with a plastic knife [20]. Fruit juices and wine samples were diluted with distilled water and the proposed assay was directly applied.

### 2.3. Preparation of CEW‒AgNC based prooxidant biosensor

Silver nanoclusters were prepared after slight modifications as described in our previous study [20]. Briefly, protein solution (50 mL, 10 mg mL^‒1^) was added to AgNO_3_ solution (50 mL, 2.5 mM) and vortexed for 2 min before the addiction of NaOH solution (12.5 mL, 5.0 N). The final mixture was incubated in a 37 °C shaken water bath for 20 h. After this time, the color changed from white to yellow, and the synthesized CEW−AgNCs were stored in the refrigerator at 4 °C before use.

### 2.4. Determing prooxidant activity using CEW‒AgNC biosensor

One mL of CEW−AgNC, 1 mL of phosphate buffer, 0.5 mL of Cu(II) solution, (x) mL of standard antioxidant or sample solution, and (1−x) mL of distilled water were added to a test tube. After the incubation for 15 min at room temperature, 0.5 mL EDTA and 1 mL Nc solutions were added and incubated for 20 min. At the end of this time the absorbance values were recorded at 450 nm against reagent blank including all solutions except the analyte. First incubation period was required to reduce cupric to cuprous by phenolic compounds. Second incubation period was required to remove free cupric ions remaining in the solution, and to break the bond of protein−bound (Cu) to obtain Cu(I)−Nc chelate with neocuproine reagent. The calibration equations were calculated by using the graphs plotted between concentration versus absorbance for each compound and the molar absorption coefficients were figured out from the slope of the calibration line concerned.

### 2.5. Determining prooxidant activity using carbonyl assay

The carbonyl assay is based on the measurement of the absorbance of the dinitrophenylhydrazone (DNP) compound formed as a result of the reaction of the 2,4‒DNPH reagent with the carbonyl groups released as a result of protein oxidation [23,24]. This assay was applied with slight modification similarly to Akyuz’s study [19], CEW−AgNC solution was incubated with phosphate buffer, Cu(II), standard antioxidant or sample, and DNPH solutions for 30 min at room temperature. After this time, absorbance measurements were recorded at 370 nm against reagent blank.

### 2.6. Statistical analysis

All experiments were performed in triplicate. Excel software (Microsoft Office 2016) was used for calculating the mean and the standard error of the mean {mean ± standard deviation (SD)}. The *F*−test was used to compare the precisions of both methods.

## 3. Results and discussion

This study was the green synthesized method using chicken egg white proteins that was carried out with slight changes in the synthesis method of our previous study [20] and applied for the first time to measure the copper-induced prooxidant activities of phenolic antioxidants as prooxidant biosensor. A novel CEW−AgNC prooxidant biosensor was proposed to measure copper catalyzed prooxidant activities of CAT, ECAT, EGCG, RES, GA, CLA, and RT as standard phenolic compounds, fresh fruits, commercial fruit juices, and red wine. The developed assay involved the reduction of cupric ions by the phenolic antioxidants and binding of cuprous ions to *S*‒terminal residues of protein in the silver nanoclusters inducing site‒specific protein damage via reactive oxygen species (ROS) formation. The prooxidant activities of phenolic compounds were indirectly determined by measuring protein bound‒Cu(I) known as a marker of prooxidant activity, utilizing ethanolic Nc reagent to obtain Cu(I)‒Nc chelate. 

### 3.1. Optimization of CEW‒AgNC synthesis conditions

For optimizing synthesis conditions, firstly concentration of phosphate buffer solution that was used for preparing protein solution before the synthesis of CEW‒AgNC was studied. Within this experiments, 0.1, 0.2, and 0.5 M phosphate buffer solutions were used for preparing protein solution which was used for synthesis of NC, and maximum absorbances of synthesized NC solutions were achieved with 0.2 M phosphate buffer. Secondly, concentration of AgNO_3_ solution (0.45 mM, 2.25 mM and 0.35 M) was studied and the best results were obtained at 2.25 mM AgNO_3_ concentration. Although, a 6:1 ratio between nucleic acid and silver concentrations was mentioned in DNA‒stabilized AgNC synthesis in the literature [8,25], this ratio was not suitable for those prepared with protein. Finally, pH values of synthesized CEW‒AgNC solutions were evaluated between 11‒13 and the highest absorbance values were obtained at pH 13.

### 3.2. Optimization of incubation periods

**Figure 1:**
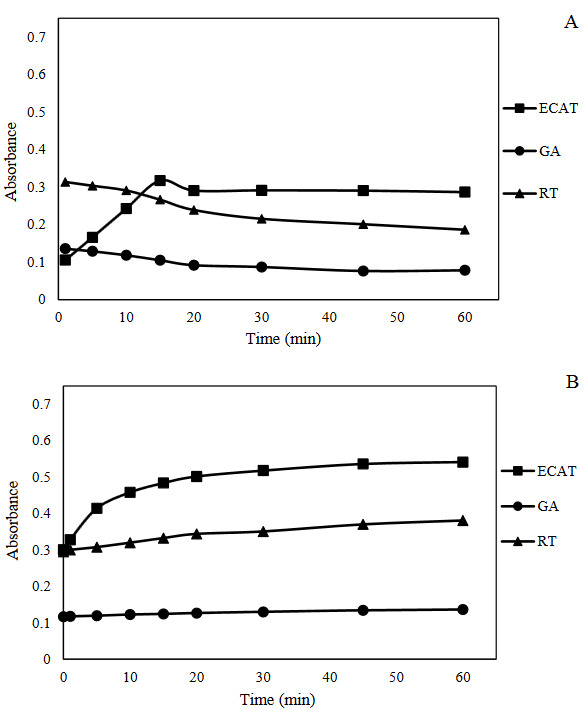
Optimization of time periods of the proposed assay with epicatechin (ECAT), gallic acid (GA), and rutin (RT) at 40 μM of each standard.

The developed assay involved two incubation periods, that the first incubation was required for the reduction of cupric ion, and binding to the protein thiol in the CEW‒AgNC (Figure 1A). The second incubation was required to eliminate excess Cu(II) with EDTA and separating the bond between copper and protein using neocuproine reagent to obtain Cu(I)‒Nc chelate (Figure 1B). To optimize these periods, time intervals from 1 to 60 min was applied to epicatechin (ECAT), gallic acid (GA), and rutin (RT) compounds at equal concentrations (40 µM) with triple experiments. As can be seen in Figures 1A and B, 15 and 20 min for first and second incubation periods respectively were found more appropriate for the three tested compounds.

### 3.3. Analytical performance of the proposed biosensor

**Figure 2:**
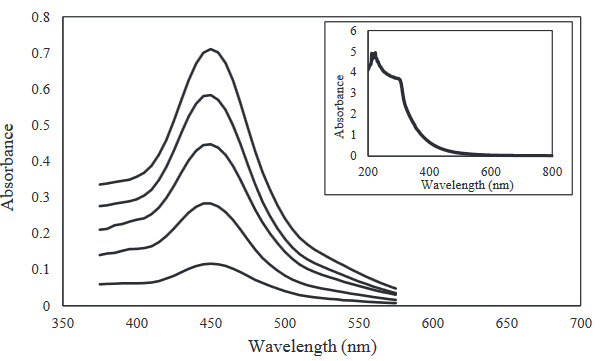
Spectra of epicatechin between 10‒50 μM concentrations. Inset: absorption spectra of synthesized CEW‒AgNC solution.

Absorption spectra of epicatechin between 10‒50 μM concentrations with respect to the proposed biosensor and absorption spectra of synthesized CEW‒AgNC solution were given in Figure 2. Validation parameters of the proposed biosensor were studied and the results obtained were summarized in Table 1. Using the slope of the calibration line of the epicatechin standard and standard deviation of the blank, limit of detection (LOD) and limit of quantification (LOQ) values were calculated. According to the calculations, the LOD and LOQ values which were the lowest values in the literature, were found to be 0.2 and 0.7 μM ECAT equivalent, respectively. CEW‒AgNC biosensor was found more sensitive than CEW‒AuNC biosensor, which was reported that AgNCs were more reactive than their gold analogs [9,16]. Accuracy of the proposed biosensor was determined by evaluating percent recovery values of wine, grape, and apple samples that spiked at three different concentrations 10, 20, 30 μM ECAT standard. The obtained recovery values were 98.4, 97.2, 98.9 for wine; 98.6; 98.2; 97.9 for grape; and 98.1; 98.7; 98.9 for apple, respectively. Intra-day precision was studied with three different experiments in a day and inter-day reproducibility was studied at three different days by determining epicatechin standard at 20 μM concentration level. The obtained relative standard deviation values were 0.96 and 1.91, respectively. All experiments were repeated three times.

**Table 1:** Validation parameters of the CEW‒AgNC biosensor for determining prooxidant activity using UV‒vis spectrophotometer.

Parameter	Value
Calibration equation	A = 12885 c – 0.013
Correlation coefficient (r)	r = 0.9999
Calibration range (μM)	0.7–50
Limit of detection (μM)a	0.2
Limit of quantification (μM)a	0.7
Accuracy – spike in wine (recovery, %) (n = 3)b	98.4; 97.2; 98.9
Accuracy – spike in grape (recovery, %) (n = 3)b	98.6; 98.2; 97.9
Accuracy – spike in apple (recovery, %) (n = 3)b	98.1; 98.7; 98.9
Intra‒day precision (RSD, %) (n = 3)c	0.96
Inter‒day reproducibility (RSD, %) (n = 3)c	1.91

a LOD = 3sbl/m (where m is the slope of the calibration line, and sbl is the standard deviation of the blank), aLOQ = 10sbl/m,b Accuracy was determined by evaluating recovery values of wine, grape and apple samples that spiked at 10, 20, 30 μM epicatechin standard.c Intra‒day precision was studied with three different experiments in a day and inter‒day reproducibility experiments were studied at three different days by determining epicatechin standard at 20 μM concentration level.

The statistical comparison of CEW‒AgNC biosensor and carbonyl assay were performed by appling the *F*‒test at 95% confidence level and the TPAs of epicatechin and red wine (10 fold diluted) were expressed as mg ECAT L^−1^ equivalents. There was no significant difference at 95% confidence level between both methods with respect to the *F*‒test data presented in Table 2.

**Table 2:** Statistical comparison of TPA of epicatechin and red wine (10 fold diluted) using F‒test at 95% confidence level with respect to the both assays.

Sample	Parameter	CEW‒AgNC biosensor		Carbonyl assay
ECAT standard	No. of samples	5		5
	Average TPA	144.29a		141.21a
	SDb	1.16		1.60
	Variance	1.34		2.56
	Degrees of freedom		4	
	Fcalculated		1.91	
	Fcritical		6.39	

Red wine	No. of samples	5		5
	Average TPA	45.37a		54.53a
	SDb	1.11		1.79
	Variance	1.23		3.20
	Degrees of freedom		4	
	Fcalculated		2.60	
	Fcritical		6.39	

a mg ECAT L−1 equivalent.b Standard deviation.

Calibration equation, correlation coefficient (r) and linear range (µM) values of some natural antioxidant standards were given at Table 3. As shown in the Table 3, excellent linearity was obtained between absorbance and concentration with respect to the proposed biosensor for all compounds. All correlation coefficient values were found above *r *= 0.999. The order of molar absorption coefficients of the studied compounds by using the CEW‒AgNC was as follows: ECAT > RT > CAT > RES > EGCG > CLA > GA. 

**Table 3:** Calibration equation, correlation coefficient (r) and linear range (µM) of some natural antioxidant standards using CEW‒AgNC biosensor.

Compound	Calibration equation	Correlation coefficient (r)	Linear range (µM)
ECAT	A = 12885 c – 0.013	r = 0.9999	0.7–50
CAT	A = 7928 c – 0.005	r = 0.9998	20–100
EGCG	A = 3140 c + 0.018	r = 0.9997	20–100
RES	A = 3505 c + 0.005	r = 0.9998	20–100
GA	A = 1676 c + 0.033	r = 0.9998	20–100
CLA	A = 2081 c + 0.029	r = 0.9997	20–100
RT	A = 9868 c + 0.023	r = 0.9993	20–100

The molar absorptivity of ECAT was found higher than CAT and other phenolic acids and this result was supported similarly with one of our previous works [19]. On the other hand, in the study of Kondakçı et al. [18], the order of prooxidant activity of tea catechins was found similar as ECAT > CAT > EGCG with respect to the molar absorption coefficients. Also it was supported with the literature as the prooxidant activity of RES was lower than catechins and higher than phenolic acids [20]. Rice‒Evans et al. [26] stated that the polyphenols with the *o*‒dihydroxy structure in the B ring included the highest scavenging activities. They also reported that under physiological conditions flavonoids which had the half peak reduction potencial values below <0.06 mV, underwent redox cycles and were thus might be reduce transition metals. Eghbaliferiz and Iranshahi [27] stated that RES exhibit prooxidant activity in the presence of copper. In the study of Ahmad et al. [28], resveratrol caused mutations to the plasmid DNA. They treated the DNA samples with increasing concentrations of RES (10‒200 µM) in the presence and absence of copper ions and said that resveratrol was act as prooxidant at higher concentrations. Zhen et al. [29] studied the prooxidant activity of some hydroxycinnamic acid compounds, measuring DNA damage in the presence of Cu(II) ions. They found that the prooxidant activity of the compounds bearing *o*‒dihydroxyl group such as CLA were displayed much higher than the others which had no functional groups. Simić et al. [30] examined the electrochemical oxidation of some phenolic compounds with cyclic voltammetric method. They reported that the compounds with low anodic potential (Epa) (<0.45 V) demonstrated antioxidant activity, whereas the compounds with high Epa values (>0.45 V) acted as prooxidants. In their study, RT behaved as an antioxidant with the 0.23 V Epa values. Yang et al. [31] investigated the DNA damage caused by quercetin, rutin, *p*‒coumaric acid and their derivatives with 300 µm concentration. In their study, it was found that rutin had higher prooxidant activity than phenolic acids. Lambert and Elias [32] reported that green tea catechins such as CAT, ECAT, and EGCG, might be responsible for the induction of apoptosis in tumor cells, although they were known as antioxidant compounds. Furthermore, in the study of Zhou and Elias [33] it was mentioned that EGCG showed higher prooxidant activity with low pH of solution medium and they detected lower prooxidant activity with the higher pH values. This information was supported with the iron(III)-induced prooxidant activity assay worked in pH 5.5 and found the highest prooxidant activity value of EGCG in the literature, instead of the other assays worked in physiological pH [22].

Comparison of the proposed method with previously published methods in terms of limit of detection (LOD) values as μM ECAT equivalent was shown in Table 4. It can be seen that the lowest LOD value in the literature was achieved with the proposed biosensor.

**Table 4:** Comparison of the proposed method with previously published methods in terms of limit of detection values as μM ECAT equivalent.)

Method	Detection	LOD (μM)	Reference
CEW‒AgNC	UV‒vis	0.2	Present work
CEW‒AuNC	Fluorescence	0.7	[21]
CEW‒AuNC	UV‒vis	0.9	[20]
Solid protein based Cu(I)‒Nc assay	UV‒vis	1.2	[19]
Solid protein based Fe(II)‒Fz assay	UV‒vis	0.5	[22]
Modified carbonyl assay (using solid protein)	UV‒vis	3.0	[19]
Chromatographic analysis	HPLC‒DAD	1.9 (μg/mL RT eq.)6.5 (μM ECAT eq.)	[34]
BSA‒AgNC	Fluorescence	0.8 (μM Cys eq.)1.9 (μM ECAT eq.)	[16]
DNA‒CuNC	Fluorescence	2.0 (μM Cys eq.)4.8 (μM ECAT eq.)	[35]

### 3.4. Total prooxidant activities of real samples

**Figure 3:**
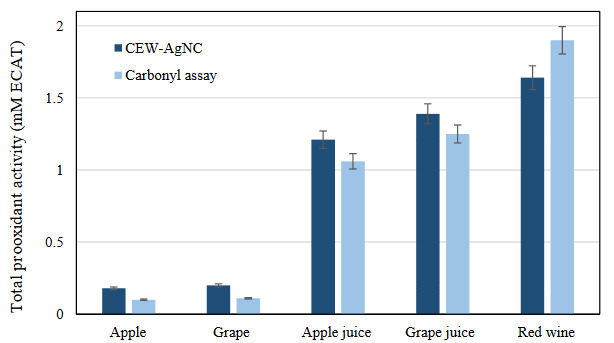
Comparison of TPA values of apple, grape, apple juice, grape juice, and red wine expressed as mM epicatechin equivalent with respect to the both CEW‒AgNC and carbonyl assays.

The primary components of the samples were as follows: chalcones, flavanols, flavonols, and condensed tannins in apple and apple juice [36]; anthocyanins, flavanols, flavonols, phenolic acids in grape, and grape juice [37]; anthocyanidins, flavanols, flavonols, and especially resveratrol in red wine [28,36,37], respectively. TPA values of fresh apple and grape, commercial apple and grape juices, and red wine were determined as mM epicatechin equivalent and compared with those of carbonyl assay as reference method. As you can see in Figure 3 the results were compatible with each other.

In the study of Rice‒Evans et al. [26], it was indicated that the amount of epicatechin was approximately 55 times higher than resveratrol in red wine. Since red wine contains strong prooxidant compounds such as catechin derivatives and resveratrol, higher TPA was determined than apple and grape samples. TPA of the apple containing phenolic acid derivatives was found to be low due to relatively low prooxidant ability of related compounds. 

In the evaluating Figure 3, the lowest TPA value was found in fresh fruits and so it can be concluded that consuming fresh fruit is healthier than consuming fabricated fruit juices. In this context, in order to be protected from various diseases and to live healthy, all fabricated products and prepackaged foods should be refrained and taken care to consume fruits and vegetables in the most natural form as much as possible.

## 4. Conclusion

Phenolic bioactive compounds known as health-beneficial antioxidants are able to exhibit prooxidant behaviour causing to increase oxidative process exponentially depending on high concentration, high pH, presence of high free transition metal ion and biological material in the medium. Thus, the determination of prooxidant activity of phenolics has gained importance as it may guide to diets and the treatment of various diseases. There are rare prooxidant biosensors in the literature to determine the prooxidant activities of natural antioxidants through protein damage. The aim of this study was to develop a novel, low cost, easily applicable, fast, sensitive, and reproducible biosensor determining the prooxidant activity in contrast to the methods in the literature, which were expensive, time-consuming, and require qualified personnel like ESR. In this study, spectrophotometric silver nanocluster based prooxidant biosensor, prepared by using chicken egg white protein was synthesized in the principle with green chemistry and applied as a prooxidant biosensor for the first time to detect copper catalyzed prooxidant activity of phenolic antioxidants abundant in foods, beverages, cereals. Protein was utilized as both reducing and stabilizing agent in the silver nanocluster synthesis procedure. In the assay, after phenolic antioxidants reduced cupric ions, cuprous ions were bound to the protein *S*‒terminal residues on the CEW‒AgNC surface inducing site‒specific protein damage via ROS formation. Protein bound‒Cu(I), as a marker of prooxidant activity, was precisely determined via CEW‒AgNC biosensor with the lowest LOD value (0.2 µM) in the literature using neocuproine (Nc) reagent. Accuracy was evaluated with recovery values obtained up to 100% that spiked at three different concentrations of ECAT standard. Precision and reproducibility studied by determining ECAT standard as intra-day and inter-day experiments were found 0.96% and 1.91%, respectively. The TPA values of fresh fruits, commercial fruit juices, and red wine were calculated and the results were statistically compared with those of carbonyl assay. The most important advantage of using nanoclusters in medicine especially in cancer diagnosis and treatment is their ability to penetrate to the kidney tissue because of their ultrasmall size and to easily dissipate from the body to reduce toxicity in vivo unlike nanoparticles [38]. In addition, considering the results of the proposed method, it was thought to give important ideas in terms of healthy life and nutrition with the controlling of oxidative stability of fabricated products and additives that had extended shelf life.

## CRediT authorship contribution statement

The corresponding author is responsible for all contributions.

## Funding

This research did not receive any specific grant from funding agencies in the public, commercial, or not-for-profit sectors.
